# Isothermal Diagnostic Assays for Monitoring Single Nucleotide Polymorphisms in *Necator americanus* Associated with Benzimidazole Drug Resistance

**DOI:** 10.1371/journal.pntd.0005113

**Published:** 2016-12-08

**Authors:** Nour Rashwan, Catherine Bourguinat, Kathy Keller, Nipul Kithsiri Gunawardena, Nilanthi de Silva, Roger Prichard

**Affiliations:** 1 Institute of Parasitology, Macdonald College, McGill University, Ste Anne de Bellevue, QC, Canada; 2 Department of Parasitology, Faculty of Medicine, University of Kelaniya, Ragama, Sri Lanka; University of Cambridge, UNITED KINGDOM

## Abstract

**Background:**

Soil-transmitted helminths (STHs) are the most prevalent intestinal helminths of humans, and a major cause of morbidity in tropical and subtropical countries. The benzimidazole (BZ) drugs albendazole (ABZ) and mebendazole (MBZ) are used for treatment of human STH infections and this use is increasing dramatically with massive drug donations. Frequent and prolonged use of these drugs could lead to the emergence of anthelmintic resistance as has occurred in nematodes of livestock. Previous molecular assays for putative resistance mutations have been based mainly on PCR amplification and sequencing. However, these techniques are complicated and time consuming and not suitable for resource-constrained situations. A simple, rapid and sensitive genotyping method is required to monitor for possible developing resistance to BZ drugs.

**Methods:**

To address this problem, single nucleotide polymorphism (SNP) detection assays were developed based on the Smart amplification method (SmartAmp2) to target codons 167, 198, and 200 in the β-tubulin isotype 1 gene for the hookworm *Necator americanus*.

**Findings:**

Diagnostic assays were developed and applied to analyze hookworm samples by both SmartAmp2 and conventional sequencing methods and the results showed high concordance. Additionally, fecal samples spiked with *N*. *americanus* larvae were assessed and the results showed that the *Aac* polymerase used has high tolerance to inhibitors in fecal samples.

**Conclusion:**

The *N*. *americanus* SmartAmp2 SNP detection assay is a new genotyping tool that is rapid, sensitive, highly specific and efficient with the potential to be used as a field tool for monitoring SNPs associated with BZ resistance. However, further validation on large numbers of field samples is required.

## Author Summary

Hookworms are amongst the major STHs and the second most prevalent intestinal helminth of humans. Large-scale treatment with the benzimidazoles (BZs) albendazole or mebendazole is the major control strategy against STHs in mass drug administration (MDA) programs. Prolonged and repeated treatment with the same anthelmintics has led to the emergence of widespread BZ resistance in veterinary parasites which is caused by a single nucleotide polymorphism at codon 200, 167 or 198 in the β-tubulin gene. There is a considerable concern that prolonged use of the same anthelmintics with suboptimal efficacy against hookworms, may select for resistant parasites and favour the development of resistance. We developed a novel genotyping assay to screen for β-tubulin polymorphisms in *N*. *americanus*, using the SmartAmp2 method. SmartAmp2 is a unique genotyping technology that detects a mutation under isothermal conditions with high specificity and sensitivity. The *N*. *americanus* SNP detection assay is rapid, sensitive and highly specific and has the potential to be used in the field for the detection of SNPs associated with BZ resistance.

## Introduction

Intestinal helminths cause a major burden on human health in developing countries, infecting more than 2 billion people worldwide [1]. Hookworms are one of the major STHs and the second most prevalent intestinal helminth of humans [2], infecting an estimated 438.9 million people in resource-constrained countries in the tropics and subtropics [3], and causing 22.1 million disability-adjusted life years [4]. Maternal hookworm anemia can complicate pregnancy, placing both mothers and children at higher risk of mortality. Infected children are compromised with anemia, stunted growth, and physical and intellectual growth deficits [5, 6]. Hookworm infections are mainly caused by *N*. *americanus* and *Ancylostoma duodenale*, with *N*. *americanus* being the most prevalent species, representing ~85% of all hookworm infections, and are associated with more morbidity worldwide than any other STH [7].

Current efforts to control morbidity caused by hookworms rely heavily on large-scale administration of ABZ or MBZ in MDA programs [8] which have been greatly expanded in recent years by massive donations of these anthelmintics. However, a single dose of either drug shows suboptimal efficacy against hookworms [9–13]. Treatment with these drugs is the major hookworm control strategy recommended by the World Health Organization (WHO) [14, 15] as there is no vaccine available. A major concern is that MDA over prolonged periods using the same anthelmintics would exert selection pressures on hookworm parasite populations and favour the development of resistance [8, 16]. In veterinary nematodes, BZ resistance has been developed and is caused by a single nucleotide polymorphism (SNP) in the β-tubulin isotype 1 gene that substitutes tyrosine (Tyr) for phenylalanine (Phe) at codon 167 or 200 (TTC>TAC) or alanine (Ala) for glutamate (Glu) at codon 198 (GAG>GCG) [17–21].

Benzimidazole resistance-associated mutations have been found in many veterinary nematodes particularly in nematodes in the same phylogenetic clade as hookworms [12, 22]. Such SNPs have already been observed in *N*. *americanus* and *Trichuris trichiura* [23, 24]. Furthermore, the presence of resistance-associated SNPs at codon 200 and 198 increased with treatment and were significantly higher in individuals who showed a poor response to ABZ (low efficacy) than in individuals who responded well to ABZ (good efficacy) in *T*. *trichiura* [24]. With the possibility that BZ susceptibility could be decreased by repeated rounds of MDA, it is important to monitor the level of resistance-associated SNPs in STHs before resistance becomes clinically manifested. The significant success and expansion of MDA programs for the control of human STHs, including hookworms, increases the urgency to monitor for drug resistance [25]. In human medicine, the egg reduction rate (ERR) is the gold standard for measuring drug efficacy and detection of resistance [26]. Relying on this efficacy measure alone is likely to be insensitive for detecting the early stages of the development of resistance and may only detect resistance when resistance allele frequencies are at high levels and treatment failures have already occurred [27, 28]. Furthermore, the ERR could be inappropriately interpreted as evidence of resistance, if standards similar to those used in the guidelines for anthelminthic of the World Association for the Advancement of Veterinary Parasitology were applied [10].

Molecular-based diagnostic tools are accurate and reliable [29, 30]. Diagnostic sequencing techniques are reliable and sensitive but are lengthy, complex, and too expensive for large-scale screening programs. With the rapid development of mutation-detection methods, several PCR based techniques have been developed to identify anthelmintic resistance, such as allele-specific PCR [18, 31, 32], RFLP-PCR [33, 34], real-time PCR [2, 35–37] and pyrosequencing [24, 30]. These methods are accurate and highly sensitive; however, they are unsuitable for large-scale screening of BZ resistance due to their complexity and the need for expensive equipment. Therefore, developing a rapid, simple, and accurate molecular method for monitoring for BZ resistance, without the need for expensive equipment, is highly desirable.

Here we report the development of a novel genotyping assay to monitor for the presence or absence of β-tubulin polymorphisms in *N*. *americanus*, using the SmartAmp2 method (Smart Amplification Process). SmartAmp2 is a unique DNA amplification method for rapid detection of target DNA or genetic polymorphisms under isothermal conditions, in a single step which eliminates the need for PCR amplification or a thermocycler [38]. SmartAmp2 is similar to LAMP, but LAMP technology uses symmetrical primer design. In SmartAmp2, however, the asymmetrical primer design is a key feature responsible for the suppression of the mismatch amplification, which minimizes the free-primer hybridization, other priming events and alternative mis-amplification pathways [39].

This method can detect a SNP with high specificity and sensitivity within 30 min [38]. The SmartAmp2 method uses *Aac* DNA polymerase which has strand displacement activity, combined with asymmetric primer design and *Thermaus aquaticus* MutS (*Taq* MutS) enzyme, which give the assay high specificity [38, 39]. *Taq* MutS is a mismatch binding protein [40], which recognizes a mismatched pair between the target DNA and the discrimination primer. This protein binds to mismatched nucleotides and blocks the dissociation of mismatched DNA by the *Aac* polymerase, which inhibits further amplification from non-target DNA [39].

The aim of this study was to develop molecular genotyping assays for the detection of the three β-tubulin polymorphisms associated with BZ resistance in other nematodes and validate their specificity and reliability in human hookworm samples and in fecal samples.

## Materials and Methods

### Study approval and ethical considerations

Hookworm eggs and larvae were collected during a study conducted in Sri Lanka on the comparative efficacy of different MBZ polymorphs for the treatment of hookworm infections and molecular markers of drug resistance in hookworms. Ethical clearance for the study was granted by the Ethics Review Committee of the Faculty of Medicine, University of Kelaniya (P39/04/2010).

Ethical approval (study 2535) was also obtained by Dr. Patrick Lammie, CDC, Atlanta, GA, and included the collection and examination of fecal samples from Haiti for helminth eggs, and DNA analysis of helminth eggs. Oral informed consent was obtained from all human adult participants and from parents or legal guardians of minors, as described previously [24, 30].

### Parasite material and DNA extraction

*N*. *americanus* adult worms, larvae and eggs were available in our lab [22, 28]. Additional larvae (L3) were cultured using the Harada-Mori technique from fecal samples collected in the field in Sri Lanka. All egg and larval samples were preserved in 70% ethanol after collection. Eggs and larvae were isolated under a dissecting microscope using a 10 μl pipette. Genomic DNA was extracted from larvae and eggs as described [41]. Lysis buffer was prepared as follow (KCl [50 mM], Tris [10 mM] with pH 8.3, MgCl_2_ [2.5 mM], 0.45% Nonidet P-40, 0.45% Tween 20 and 0.01% gelatine). Ten μl proteinase-K [10 μg / ml] (Invitrogen, Life Technologies; Burlington, ON, CA) and β-mercaptoethanol (Sigma-Aldrich, ON, CA) were added to 1 ml of this buffer just before use. Twenty-five μl of lysis buffer mix was added to previously isolated eggs and larvae and then tubes were incubated at 60°C for 2 h. Genomic DNA was extracted from adult *N*. *americanus* using QIAamp DNA mini kit (Qiagen, Hilden, Germany) according to the manufacture’s protocol.

### Wild-type (WT) and mutant-type (MT) plasmid constructs

To assist with SmartAmp2 development, control plasmids were engineered by site-directed mutagenesis as previously described [23]. Extracted genomic DNA from individual adult worms was used to amplify a fragment of the *N*. *americanus* β-tubulin isotype 1 gene including the codon positions 167 (exon 4), 198 and 200 (exon 5). Specific forward primer 5’-AAGAAGCTGAAGGATGTGACTG-3’ and specific reverse primer 5’-GAAGCGA AGACAGGTAGTAACAC-3’ (Invitrogen), were designed in the exonic regions of *N*. *americanus* genomic DNA sequence (GenBank accession no. EF392851). The PCR master mix contained 2 μl 10×PCR buffer, 1 μl (50 mM) MgSO_4_, 1 μl dNTP [10mM], 1 μl of each forward and reverse primer [10 μM], 1 U Platinum *Taq* DNA polymerase High Fidelity (Invitrogen), 2 μl genomic DNA and distilled H_2_O to reach a final volume of 20 μl. Negative controls (no template) were also included for quality control. The PCR reaction conditions were 94°C for 3 min, followed by 35 cycles at 94°C for 45 s, 57°C for 45s and 68°C for 1 min and a final extension at 68°C for 10 min. The resulting PCR fragments were Sanger sequenced to confirm the presence of sensitive alleles at codon positions 167, 198, and 200. Plasmids carrying mutations at position 167, 198, or 200 (MT) were engineered by site-directed mutagenesis. Primers for MT plasmids (outer primers and inner primers carrying the mutant alleles) are shown in Table 1. Amplified WT or MT fragments were cloned into TOPO-TA-Cloning vector (Invitrogen). Plasmid DNAs were extracted and purified using QIAprep miniprep plasmid kit (Qiagen) and subsequently sequenced by Sanger sequencing at the McGill University/Genome Quebec Innovation Centre, Montreal, Quebec. The purity and quantity of DNA in clones was measured using a Nano Drop Spectrophotometer, ND-1000 (Implen, Munich, Germany). Diluted WT and MT plasmids were used for assay optimization and development, and to determine the detection limit of each assay.

**Table 1:** *N*. *americanus* specific primers for the mutant plasmid constructs *N*. *americanus* specific primers for the mutant plasmid constructs for three SNP positions in the β-tubulin isotype 1 gene

Codon	Primer sequences (5′-3′)	(°C)
167	Forward: AAGAAGCTGAAGGATGTGACTG	
	Reverse: GAAGCGAAGACAGGTAGTAACAC	58
	SNP Fwd: CATGTCCTCGT**A**TTCCGTTG	
	SNP Rev: CAACGGAA**T**ACGAGGACATG	
198	Forward: AAGAAGCTGAAGGATGTGACTG	
	Reverse: GAAGCGAAGACAGGTAGTAACAC	57
	SNP Fwd: AGATG**C**GACCTTCTGTATTGATAATG	
	SNP Rev: CATTATCAATACAGAAGGTC**G**CATCT	
200	Forward: AAGAAGCTGAAGGATGTGACTG	
	Reverse: GAAGCGAAGACAGGTAGTAACAC	57
	SNP Fwd: AGATGAGACCT**A**CTGTATTGATAATG	
	SNP Rev: CATTATCAATACAG**T**AGGTCTCATCT	

* SNP-Fwd: forward primer mutated for a single nucleotide; SNP-Rev: reverse primer mutated for a single nucleotide; (°C) primer annealing temperature.

### SmartAmp2 primer design

**Fig 1:**
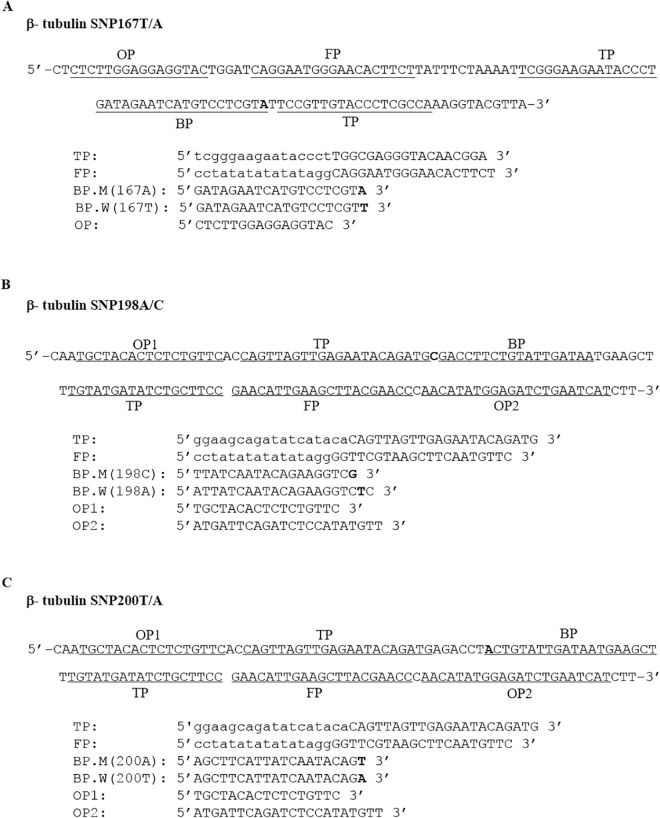
SmartAmp2 primer design for β-tubulin mutation (SNP) detection. Partial sequence of the β-tubulin isotype 1 gene carrying (**A**) SNP 167A, (**B**) SNP 198C, and (**C**) SNP 200A as well as the sequences of primers used for the SmartAmp2 assay for the three SNP positions. The locations of SNPs indicated in bold. The boost primer (BP) was used as the discrimination primer. The folding primer (FP) has a specific sequence (CCTATATATATATAGG) at the 5’ end to allow self-annealing hairpin formation.

Primer sets were designed to amplify and detect putative β-tubulin mutations in the hookworm *N*. *americanus*. The online software version 1.1 (SMAPDNA), was made available by KK. DNAFORM, Japan (http://www.dnaform.jp/en/), and used initially to design the primers. Several primer sets were suggested by the software and further refinement in primer design was made by trials and evaluation tests and the best candidate primer set was selected for each assay. A primer set consists of five specific primers, the folding primer (FP), turn-back primer (TP), boost primer (BP), and two outer primers (OP1 and OP2), designed to recognize six different sequences on the target sequence. A set of primers has two discrimination primers, specific for either the WT or MT allele, differing only in one nucleotide at the 3’-end or the 5’-end. In this work, BP primer was selected to be the discrimination primer. The location and the sequences of primers for each SNP target are illustrated (Fig 1).

### SmartAmp2 assay development and optimization

The SmartAmp2 assay was optimized using different concentrations of primers, MgSO_4,_ and betaine and carried out in 25 μl reactions containing (2–3 μM) TP/FP, (1–1.5 μM) BP, (0.25–0.4 μM) OP1/OP2 (Invitrogen) 1.4 mM dNTPs (Invitrogen), (0.8–1.4 M) betaine (Sigma- Aldrich), 1x isothermal buffer (20 mM Tris-HCl (pH 8.6), 10 mM KCl, 10 mM (NH_4_)_2_SO_4_, (4–8 mM) MgSO_4_, 0.1% Tween 20, 1/100,000 dilution SYBR Green I (Invitrogen), 1 μg *Taq* MutS (Nippon Gene, Toyama,Japan/Wako Chemicals, Richmond, VA, USA) and 12 U *Aac* DNA polymerase (KK. DNAFORM, Yokohama, Japan). Control plasmids corresponding to WT or MT alleles were used to develop each assay and to evaluate the accuracy of genotyping between different primer sets. One microliter of WT or MT plasmids (~10 ng) was heated at 95°C for 3 min before being added to the assay. Reactions were incubated at 60°C for 60 min. The Rotor-Gene Q system (Qiagen) was used to maintain isothermal conditions and to monitor the change in fluorescence intensity of the intercalating SYBR Green I during the reaction. Assays were evaluated in terms of amplification (full match) and non-amplification (mismatch) within 60 min.

### SmartAmp2 assay sensitivity and specificity

Further optimization was performed to estimate the sensitivity and reproducibility of the assays in individual samples and pools. Assays were tested on individual eggs/larva and pools (10–20 eggs/larva per pool). After DNA extraction from eggs and larvae using lysis buffer and proteinase K, 3 μl of this crude lysate was added to each reaction and then tubes were incubated at 60°C for 90 min. For evaluation of the sensitivity and the specificity of the assay to detect MT alleles in a background of WT DNA, MT plasmid DNA (~5 ng) was mixed with WT plasmid (~5 ng) in serial dilutions of 1:1, 1:9, 1:99 and 0: 100 and assays were carried out using the MT detection primer sets. These experiments were repeated twice and each DNA sample was analysed in duplicate.

### Validation of the assay on *N*. *americanus* larval samples

Validation on field samples was performed using 110 individual samples obtained from Sri-Lanka. Pools of 20 larvae previously collected under microscopy for each individual sample were analyzed. Larval samples were digested using 25 μl of previous lysis buffer mix. From this crude lysate, 3 μl were added to each reaction after a DNA heating step at 95°C for 3 min. Assays were carried out in 25 μl reactions as previously explained. Positive (adult worm genomic DNA) and negative controls were always included as a reference in each experiment. Tubes were incubated in a real-time PCR at 60°C for 90 min.

### Genotyping of *N*. *americanus* larvae using PCR sequencing method

PCR amplification of β-tubulin gene around codon positions 167, 198 and 200 were performed. Primers were designed as follow. For codon 167, forward and reverse primers were respectively 5’-AAGAAGCTGAAGGATGTGACTG-3’ and 5’-GGGTGGTTCCAGGCT GATGC-3’. For codons 198/200 (exon 5), forward and reverse primers were respectively, 5’-GGTTTCCGACACTGTGGTTG-3’ and 5’-GAAGCGAAGACAGGTAGTAACAC-3’. The PCR master mix contained 5 μl 10×PCR buffer, 1.25 μl (50 mM) MgSO_4_, 1 μl dNTP [10mM], 1 μl of each forward and reverse primer [10μM] (Invitrogen), 1 U Platinum *Taq* DNA polymerase High Fidelity, 3 μl of each DNA sample and distilled H_2_O up to 50 μl. The PCR reaction conditions were 94°C for 4 min, followed by 35 cycles at 94°C for 45 s, 57°C for 45 s and 68°C for 1 min and a final extension at 68°C for 5 min using a thermocycler (Biometra, Göttingen, Germany). Amplicons were identified on 2% agarose gel and visualized under UV (Bio-Rad Molecular Imager Gel Doc XR System). PCR products were sent to McGill University/Genome Quebec Innovation Centre, Montreal, Quebec for conventional Sanger sequencing to confirm and validate the genotyping results previously obtained with SmartAmp2 assay. Electropherograms were analyzed with Sequencher software (version 4.10.1) to identify the genotypes.

### Fecal sample assessment by SmartAmp2 assay

To validate the specificity of the assay on DNA extracted from fecal samples, genomic DNA was extracted from parasite-free fecal samples spiked with *N*. *americanus* larvae (~1000), or was extracted from the same fecal samples (with no larvae added) and used as a negative fecal control. As a positive control, DNA was extracted from purified L3 larvae (~1000). A protocol of QIAamp fast DNA stool mini kit (Qiagen) was used with some modifications. The fecal suspension (~ 1g) was kept on at 30°C to evaporate the ethanol. Then the InhibitEX buffer (QIAamp kit) was added together with approximately 150 mg of 0.5 mm glass beads (Sigma-Aldrich) and the sample vortexed on a Vortex-Genie 2 at 3000 rpm for 3 cycles of 5 min each, to disrupt the cell wall of the helminth samples. The fecal suspensions were then heat-shocked at 95°C for 5 min and placed in liquid nitrogen for 2 min, for 5 cycles. Then the proteinase K and AL buffer (QIAamp kit) were added and heated at 60°C for 1 h. The suspension was centrifuged at 14,000 rpm for 1 min and the procedures outlined in the QIAamp kit were then followed. Three μl of the spin column eluate (100 μl) was taken for each SmartAmp assay. The reaction mix for the SmartAmp assay was prepared and carried out as previously mentioned. Negative controls were used in all experiments.

## Results

### Optimization of SmartAmp2 method for SNP detection

Various sets of candidate primers were designed to genotype the β-tubulin gene at the three SNP positions. Screening of these primer combinations and assay conditions yielded an ideal primer set for each assay, which completed the amplification within 20–30 min from the target DNA sequence in a plasmid. Primer sets were chosen based on first, the speed and yield of the amplification, and second, the primer efficiency to discriminate between the full match and mismatch amplification. In the initial optimization of the assay and without the inclusion of *Taq* MutS, a 15 min delay for the mismatch amplification was achieved. With the inclusion of *Taq* MutS, a complete suppression of the mismatch amplification was observed up to 60 min (S1 Fig). All primer sets that displayed late full match amplification or a short delay between the full-match and mismatch amplification were omitted. The location and sequences of primers for each SNP target are shown (Fig 1). The SNP 167T/A (Phy167Tyr) occurs in exon 4 of the β-tubulin gene. A set of primers was designated TP, FP, BP, and OP2. The 5’-end of the BP discriminates the polymorphism 167T or 167A. In this assay, one of the outer primers was omitted to avoid the design of primers in the intron region. As the assay is highly specific, any polymorphism in the intron regions could affect the reproducibility of the assay.

Both 198A/C SNP (Glu198Ala) and 200A/T SNP (Phy200Tyr) reside on exon 5 of the β-tubulin gene. One set of primers was designed (TP, FP, OP1 and OP2) for both assays but a specific BP (discrimination primer) was designed in which the 3’-end of 198BP or 200BP discriminates the polymorphism at 198A/C or 200A/T, respectively. For each assay, BP primers were designed to be specific either for a MT variant or a WT allele. This unique primer design for SNP 198 and 200 makes reaction setup simple and easy to perform within a short time for a large number of samples.

**Fig 2:**
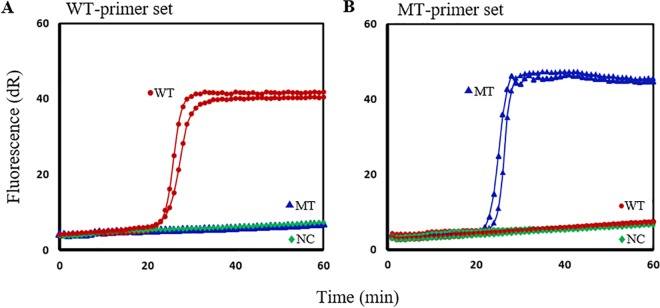
SmartAmp2 assay optimization for *N*. *americanus* β-tubulin SNP at codon 200T/A. (**A**) Wild-type (WT)-specific primer amplification of WT plasmid (red circle)and no amplification with mutant-type (MT) plasmid (blue triangle) or NC (no template) (green diamond). (**B**) MT-specific primer amplification of MT plasmid (blue triangle) and no amplification with WT plasmid (red circle) or NC (green diamond). Assays were run in duplicate.

Before testing the assay on hookworm samples, constructed WT and MT plasmids were used as DNA templates for assay optimization and development. Sequencing the MT plasmids revealed that the desired mutations at codons 167T/A, 198A/C and 200T/A of the β-tubulin gene were generated. The optimal amplification results were obtained when the reaction mixture contained 2 μM each TP/FP, 1 μM BP and 0.25 μM OP1/OP2 with 0.8 M betaine and 8 mM MgSO_4_. Primer sets with a specific BP for detecting the 167T/A, 198A/C, or 200T/A point mutations rapidly amplified the MT plasmid within 20–30 min, whereas the same primer sets failed to amplify the WT plasmid within 60 min. The WT primer sets amplified the WT plasmid but not the MT plasmid. Each assay was run in duplicate and all negative control reactions included in the experiments showed no amplification. These results confirmed that the SmartAmp2 assays were optimized as they accurately discriminated the full match amplification from the mismatch with complete suppression of the mismatch amplification. As an example, Fig 2 shows 2 different assays using a WT primer set (2A) and a MT primer set (2B) for the 200T/A β-tubulin SNP.

### Sensitivity and specificity of the SmartAmp2 SNP detection assay

**Fig 3:**
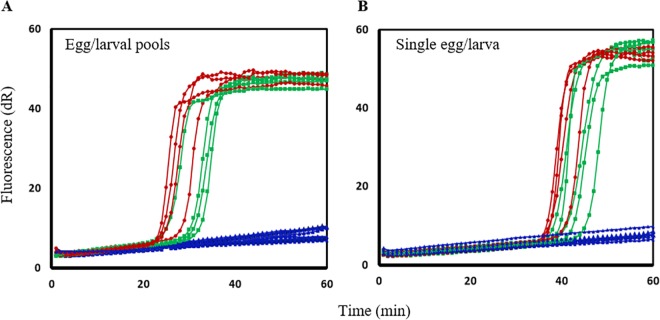
Evaluation of SmartAmp2 SNP detection assay in pools and individual hookworm samples of eggs or larvae. (**A**) Wild-type (WT)-specific primer amplification of gDNA from egg and larval pools with complete suppression of amplification using MT-specific primers. (**B**) WT-specific primer amplification of gDNA from single eggs and larva with complete suppression of amplification using the MT-specific primers. Amplification with WT primers, larva(e) (red circle), egg(s) (green square), and amplification with MT primers (blue triangle), for egg(s) or larva(e).

Further optimizations on single eggs/larva and pools of eggs or larvae were performed and full-match amplification using the WT primer sets was achieved with complete suppression of the mismatch amplification when the MT primer sets were used. Our assays amplified and genotyped DNA from single eggs and larva with high sensitivity and specificity within 40–50 min (Fig 3B). The WT primer set also amplified DNA from pools of eggs/larvae within 30–40 min with complete suppression of the amplification when the MT primer set was used (Fig 3B). The SmartAmp2 genotyping results and the Sanger sequence were always consistent.

**Fig 4:**
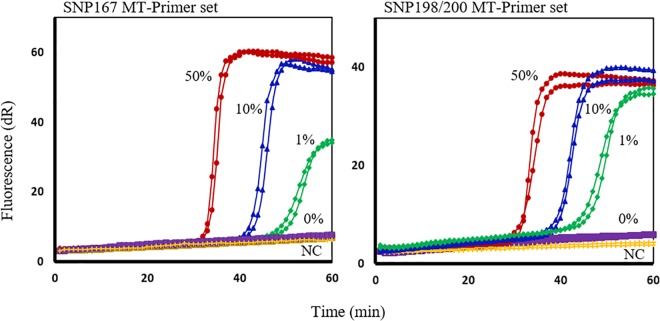
SmartAmp2 assay specificity of mutant-type (MT) allele detection in the presence of wild-type (WT) DNA. SmartAmp2 amplification for SNPs at codon 167 (first panel), and codons 198 or 200 (second panel), using the MT-specific primers with a mixture of MT plasmid and WT plasmid, 1:1 (50%; red circle), 1:9 (10%; blue triangle), 1:99; (1%; green diamond), and 0:100 (0%; purple square). Duplicate assays.

To determine the specificity of each assay for detection of the MT alleles in a mix with WT DNA, serially diluted plasmids representing MT alleles at codon 167, 198 or 200, and wild-type plasmids, were used. The results of SmartAmp2 assays, using full-match MT primer sets for each SNP target, are illustrated (Fig 4). The MT alleles were detected even when present at only 1% of the WT DNA approximately at 50 min for the SNP 198 and 200 assays and at 60 min for SNP 167 assay. Up to 90 min, no background amplification was observed from the WT DNA or the negative controls. These results show that the assay is highly sensitive as it allowed detection as low as 1% of the MT alleles in a mix of WT DNA and the ability to genotype single and pooled hookworm eggs and larvae was also demonstrated. High sensitivity and specificity of the assay are particularly important in screening for mutations in individual samples with low levels of STH infections.

### Genotyping of *N*. *americanus* larval samples by SmartAmp2 method

To validate the accuracy and specificity of the SmartAmp2 assays to handle field samples, we analyzed 110 individual field samples and a pool of 20 larvae per sample was examined. We detected the presence or absence of WT and MT alleles in all the samples within 30–40 min after incubation at 60°C. No background amplification (mismatch) was observed within 90 min. No amplification was observed from the negative controls., Three assays were performed for each sample to screen the three codon positions 167, 198 and 200. For codon 167 and 200, the WT primer set amplified the DNA target within 30–40 min. No amplification was observed with the MT primer set. None of the larval samples revealed significant levels of polymorphisms either at position 167 or 200. However, a polymorphism was identified at codon position 198A/C in some samples. The MT primer set for codon 198 allowed the amplification of the DNA target within 30–40 min (full match amplification).

**Fig 5:**
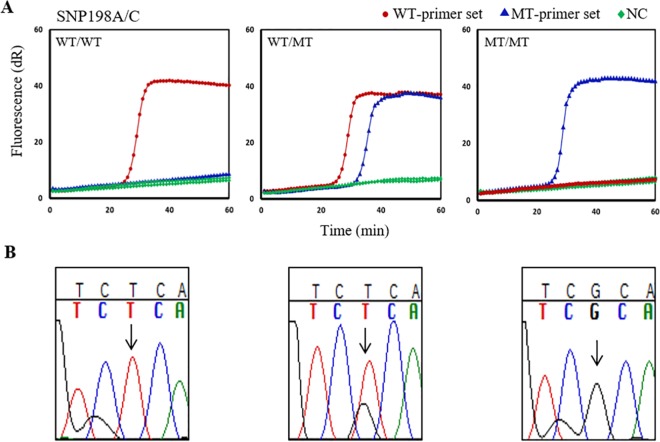
Genotyping results of *N*. *americanus* samples at codon 198 with SmartAmp2 and conventional sequencing. **(A)** SmartAmp2 amplification of 198A/C polymorphs using wild-type (WT) and mutant-type (MT)-primer sets. Left, center, and right panels show assay results for homozygous WT (WT/WT), mixed (WT/MT), and homozygous MT (MT/MT) samples, respectively. **(B)** Conventional sequencing of β-tubulin gene from left to right, WT (A/A), mixed (A/C), and MT (C/C).

The SNP198 detection primers recognized the SNP 198A/C of the β-tubulin gene to discriminate homozygous 198A/A (WT), mixed 198A/C (WT/MT), and homozygous 198C/C (MT) in genomic DNA samples. From the 110 samples examined by SmartAmp2 for the 198A/C assay, 90 samples were homozygous WT, 12 were mixed, and 8 were homozygous MT. Selected results for each of the three different genotypes using the WT and MT primer sets are illustrated (Fig 5A). The accuracy of our SmartAmp2 results was tested by amplifying the SNP target by PCR. The resultant amplicon was Sanger sequenced and showed concordance with the SmartAmp2 results (Fig 5B).

### Evaluation of SmartAmp2 assays on fecal samples

**Fig 6:**
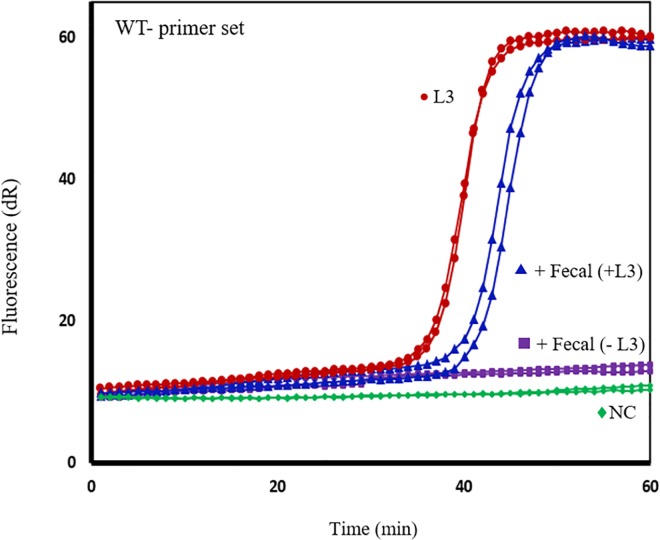
Evaluation of SmartAmp2 genotyping assays in fecal samples spiked with *N*. *americanus* larvae (L3). SmartAmp2 assay using the wild-type (WT)-primer set on DNA extracted from purified L3 (no feces) (red circle), fecal samples spiked with L3 (blue triangle) and fecal samples without L3 (purple square). Negative controls (NC; no feces, no larvae) were included in each run (green diamond).

Positive (spiked with L3) and negative fecal samples were assessed in triplicate by SmartAmp2 assay. Full-match amplification was obtained only from positive fecal samples using the WT primer set within 40–45 min. High amplification efficiency was achieved when samples were diluted 1:4 in distilled H_2_O. The negative fecal samples and the negative control remained at baseline for at least 90 min. DNA extracted from purified L3 (positive control) produced a slightly faster amplification signal than genomic DNA from spiked fecal samples (Fig 6).

## Discussion

Diagnostics are vital to achieve successful elimination of parasitic infections and to aid against emerging pathogen resistance to the limited number of anti-parasitic drugs. The low sensitivity of current field diagnostic tools could miss the early stages of resistance. The lack of rapid, simple and reliable diagnostic tools for intestinal nematodes prevents accurate estimation of the distribution of BZ-resistant populations, the determination of at-risk populations and the burden of disease [42]. In this study, we developed a new SNP genotyping assay based on the SmartAmp2 method for monitoring β-tubulin polymorphisms. Primer sets were selected and optimized specifically to target F200Y, F167Y (T**T**C/T**A**C) and E198A (G**A**G/G**C**G) SNPs in the hookworm *N*. *americanus*. SNP-detection primer sets were able to efficiently and rapidly discriminate MT and WT genotypes using plasmids as DNA templates. Assays showed high reproducibility and sensitivity for detecting genomic DNA from pools and single egg/larval DNA in a single amplification and detection step. Compared with PCR-based methods, in order to genotype single egg/larval DNA, a nested PCR using the same forward and reverse primers is required, followed by gel electrophoresis and pyrosequencing [24]; a multistep technique that is time consuming and increases the risk of contamination as a result of manipulating PCR products. Additionally, the SmartAmp2 assays showed high specificity and allowed the detection of as little as 1% of the MT alleles in a mix with WT plasmid.

Assays detected the E198A (G**A**G/G**C**G) SNP in the *N*. *americanus* larvae. The MT-detection primer set detected and identified the MT alleles in pools of larval samples from Sri Lanka. To our knowledge, this is the first time that 198SNP has been detected in *N*. *americanus*. Amplification was evident within 30–40 min using as few as 20 larvae per pool. In addition, genotyping from single larva and eggs was achieved within 40–50 min.

Fecal samples spiked with *N*. *americanus* larvae were processed in the SmartAmp2 assay and the results showed high tolerance of the *Aac* polymerase to fecal inhibitors. No significant difference in the amplification efficiency between spiked fecal extracts and purified larval DNA was observed and any slight difference could be explained by the presence of remaining fecal inhibitors in the fecal samples. SmartAmp2 assays for genotyping hookworm samples are highly sensitive and specific using the *Aac* polymerase which is tolerant to inhibitors in stool samples; however, the assay sensitivity in stool samples could be compromised by the capacity of the extraction method to obtain good quality and quantity of purified DNA, the number of eggs in stool samples particularly in individuals with low level of infection, the amount of stool used for DNA extraction and an uneven distribution of eggs in the stool. To improve the performance of SmartAmp2 assay in fecal samples, sample collection and processing should be simple and ensure high DNA yields. Preliminary enrichment of eggs through sugar flotation followed by repeated cycles of freeze/boiling to crack the egg shell and liberate DNA [43] may significantly improve DNA recovery, assay sensitivity and reproducibility. Commercial DNA extraction methods are reliable and efficient for removing PCR inhibitors present in fecal samples; however, these methods use a small amount of feces and this could affect the sensitivity of the detection assay.

SmartAmp2 assay is relatively inexpensive; the main costs are for the *Taq* MutS and *Aac* polymerase. SmartAmp2 Primers used in this study were regular primers not HPLC primers. Additionally, reducing the reaction mixture to 10 μl and using in-house prepared buffers and reagents also reduces cost. Our data were generated on a Real-Time PCR system to follow the formation of double stranded DNA in real time, using SYBR green. However, end point detection system for monitoring fluorescence that would allow high throughput analysis of samples in a 96-well microplate format could be employed. Other approaches for visualizing the formation of DNA residues could be applied using fluorescence dyes that allow colorimetric inspection of the results.

In a SmartAmp2 assay, the presence of both the WT and MT alleles can be detected in a sample, as amplification would occur with both WT and MT primer sets. The relevance would be that if the MT is detected, as well as the WT, there would be the potential for some parasites being resistant and that with further anthelmintic selection the frequency of the MT allele might increase, increasing the risk of phenotypic resistance. The absence of definitive evidence of anthelmintic resistance in human parasites does not mean that resistance alleles are not present, at least at low frequencies. Such resistance alleles could increase with prolonged and repeated selection pressure. The lack of detection of phenotypic resistance may, in part, be due to the lack of a reliable and sensitive method to monitor for resistance alleles before and after BZ treatment in control programs [44], a low frequency of resistance alleles, and the probability that BZ resistance is recessive, as it is in veterinary parasites [45].

The present study provides evidence that the SmartAmp2 method targeting β-tubulin polymorphisms in *N*. *americanus* allowed direct detection of SNPs of a target DNA sequence in fecal samples. Additionally, these results indicate that our SNP genotyping assays are rapid, simple, very sensitive and highly specific which provide a unique tool for investigating the possibility of developing BZ resistance in the hookworm *N*. *americanus*. The development of sensitive and practical methods for early detection of resistance using molecular diagnostic tools that could be adapted to the field is urgently needed in order to sustain the benefits of helminth control programs.

## Supporting Information

S1 Checklist STARD Checklist (DOCX)

S1 Fig Suppression of mismatch amplification using the mismatch binding protein *Taq* MutS Full-match amplification was achieved using the WT primer set on three WT plasmid samples (red, yellow, blue). Mismatch amplification using the MT primer set without the addition of *Taq* MutS resulted in 15 min delay (purple, green, violet) while the inclusion of *Taq* MutS in SmartAmp2 assay resulted in complete suppression of mismatch amplification.
(PDF)

S1 Flowchart STARD Flowchart (DOCX)
