# Supplementary material for: Isothermal Diagnostic Assays for Monitoring Single Nucleotide Polymorphisms in Necator americanus Associated with Benzimidazole Drug Resistance
Source: PLoS Negl Trop Dis. 2016 Dec 8;10(12):e0005113. doi: 10.1371/journal.pntd.0005113 (PMC5145137; doi:10.1371/journal.pntd.0005113)
Supplement: S1 Fig — Mismatch amplification using the MT primer set without the addition of Taq MutS resulted in 15 min delay (purple, green, violet) while the inclusion of Taq MutS in SmartAmp2 assay resulted in complete suppression of mismatch amplification. (PDF) [file pntd.0005113.s002.pdf]

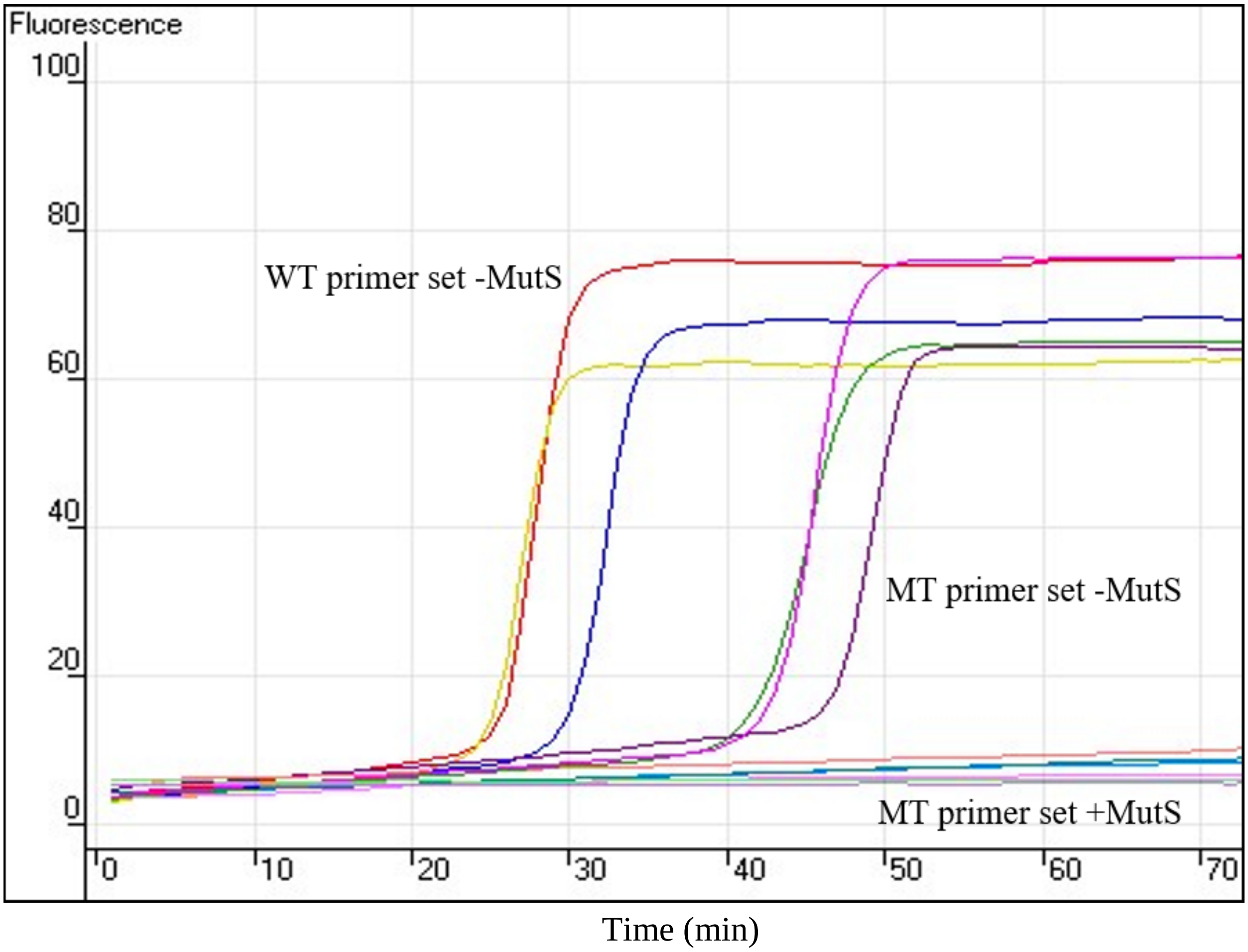

S1 Fig. Suppression of mismatch amplification using the mismatch binding protein *Taq* MutS Full-match amplification was achieved using the WT primer set on three WT plasmid samples (red, yellow, blue). Mismatch amplification using the MT primer set without the addition of *Taq* MutS resulted in 15 min delay (purple, green, violet) while the inclusion of *Taq* MutS in SmartAmp2 assay resulted in complete suppression of mismatch amplification.
