# Supplementary material for: Isothermal Diagnostic Assays for Monitoring Single Nucleotide Polymorphisms in Necator americanus Associated with Benzimidazole Drug Resistance
Source: PLoS Negl Trop Dis. 2016 Dec 8;10(12):e0005113. doi: 10.1371/journal.pntd.0005113 (PMC5145137; doi:10.1371/journal.pntd.0005113)
Supplement: S1 Flowchart — (DOCX) [file pntd.0005113.s003.docx]

**Supporting information S3. STARD flowchart.**

Index test

n=110

Final diagnosis (genotyping)

- Wild-type=90
- Mutant-type=8
- Mixed=12

Reference standard (conventional sequencing) n=85

- Wild-type=65
- Mutant-type=8
- Mixed=12

No index test

n=0

No reference standard

n=0

No reference standard

n=0

No reference standard

n=25

Index test negative

n=0

Index test positive

n=110

Index test inconclusive

n=0

Eligible participants

n=110

Potentially eligible participants

n=110

Excluded

n=0
